# Nuciferine Prevents Hepatic Steatosis and Injury Induced by a High-Fat Diet in Hamsters

**DOI:** 10.1371/journal.pone.0063770

**Published:** 2013-05-15

**Authors:** Fuchuan Guo, Xue Yang, Xiaoxia Li, Rennan Feng, Chunmei Guan, Yanwen Wang, Ying Li, Changhao Sun

**Affiliations:** 1 Department of Nutrition and Food Hygiene, Public Health College, Harbin Medical University, Harbin, Heilongjiang, P. R. China; 2 Institute for Nutrisciences and Health, National Research Council Canada, Charlottetown, Prince Edward Island, Canada; 3 Department of Biomedical Sciences, University of Prince Edward Island, Charlottetown, Prince Edward Island, Canada; University of Navarra School of Medicine and Center for Applied Medical Research (CIMA), Spain

## Abstract

**Background:**

Nuciferine is a major active aporphine alkaloid from the leaves of *N. nucifera Gaertn* that possesses anti-hyperlipidemia, anti-hypotensive, anti-arrhythmic, and insulin secretagogue activities. However, it is currently unknown whether nuciferine can benefit hepatic lipid metabolism.

**Methodology/Principal Findings:**

In the current study, male golden hamsters were randomly divided into four groups fed a normal diet, a high-fat diet (HFD), or a HFD supplemented with nuciferine (10 and 15 mg/kg·BW/day). After 8 weeks of intervention, HFD-induced increases in liver and visceral adipose tissue weight, dyslipidemia, liver steatosis, and mild necroinflammation in hamsters were analyzed. Nuciferine supplementation protected against HFD-induced changes, alleviated necroinflammation, and reversed serum markers of metabolic syndrome in hamsters fed a HFD. RT-PCR and western blot analyses revealed that hamsters fed a HFD had up-regulated levels of genes related to lipogenesis, increased free fatty acid infiltration, and down-regulated genes involved in lipolysis and very low density lipoprotein secretion. In addition, gene expression of cytochrome P4502E1 and tumor necrosis factor-α were also increased in the HFD group. Nuciferine supplementation clearly suppressed HFD-induced alterations in the expression of genes involved in lipid metabolism.

**Conclusions/Significance:**

Nuciferine supplementation ameliorated HFD-induced dyslipidemia as well as liver steatosis and injury. The beneficial effects of nuciferine were associated with altered expression of hepatic genes involved in lipid metabolism.

## Introduction

Non-alcoholic fatty liver disease (NAFLD) is increasingly diagnosed worldwide and considered to be the most prevalent liver disorder in Western countries and China [1]. NAFLD comprises a disease spectrum which includes variable degrees of simple steatosis (fatty liver), non-alcoholic steatohepatitis (NASH), and cirrhosis. Simple steatosis is benign, whereas NASH is characterized by hepatocyte injury, inflammation, and fibrosis, which can lead to cirrhosis, liver failure, and hepatocellular carcinoma. NAFLD is strongly associated with dyslipidemia, insulin resistance, obesity, and hypertension and is now regarded as a liver manifestation of metabolic syndrome [2].

*Nelumbo nucifera Gaertn* (common name, sacred lotus) is a perennial aquatic crop grown and consumed worldwide, especially in Southeast Asia, Russia, and some African countries. *N. nucifera* is utilized as an ornamental plant and a dietary staple, as well as a medicinal herb in Eastern Asia, particularly in China. The leaves of *N. nucifera* are recorded in the earliest written documentation of traditional Chinese medicinal as “Ben cao gang mu”, a medicinal herb for weight loss [3], dizziness, sunstroke, dysentery, and blood clotting [4]. Lotus leaf extracts have been shown to possess several pharmacological properties, including antioxidant [5], antiobesity [6], hypolipidemic [7], and insulin secretagogue activities [8], which have been mainly attributed the alkaloid nuciferine ((R)-1,2-dimethoxyaporphine), an aromatic ether-containing compound and a major active aporphine that was recorded as the major constituent of lotus leaf in the 2005 Chinese Pharmacopoeia [3], [9]. The main pharmacological effects attributed to nuciferine include smooth muscle relaxation, amelioration of hyperlipidemia, stimulation of insulin secretion, vasodilation, induction of hypotension, anti-arrhythmic properties, and antimicrobial and anti-HIV activities [3], [10]–[12].

Although various physiological nuciferine activities have been demonstrated, links to liver lipid metabolism have yet to be explored. Therefore, the aim of the present study was to investigate the protective effects of nuciferine supplementation on liver steatosis and injury in hamsters fed a high-fat diet (HFD). Our findings showed that the development of HFD-induced liver steatosis and injury in hamsters was significantly suppressed by nuciferine supplementation. In addition, HFD-induced alterations in the expression of hepatic genes involved in lipid metabolism, inflammation, and oxidative stress were also reversed by nuciferine supplementation.

## Materials and Methods

### Ethics Statement

All protocols in this study were approved by the Medical Ethics Committee of Harbin Medical University (Habrin, China) and were performed in accordance with the National Institutes of Health regulations for the care and use of animals in research.

### Animal Care and Experimental Protocol

Forty 6-week-old male golden Syrian hamsters (*Mesocricetus auratus*) were purchased from the Vital River Laboratory Animal Technology Company (Beijing, China) and individually housed in stainless steel cages in a room at 22±2°C on a 12-h light-dark cycle with free access to regular rodent chow and water. After 1 week of acclimatization, the hamsters were randomly divided into four experimental treatment groups (n = 10 hamsters each) as follows: group 1 was maintained on a normal diet (ND) only; group 2 was maintained on a HFD only; and groups 3 and 4 were maintained on a HFD supplemented with 10 and 15 mg/kg·body weight (BW)/day of nuciferine. The normal diet was a purified version of the AIN-93G diet with slight modifications that we previously reported [13]. The HFD was a normal diet supplemented with 10% lard and 0.3% cholesterol. The ND contained 13.9% (cal) fat and the HFD contained 33.0% (cal) fat. Diets were freshly mixed weekly in small amounts and stored at 0–4°C to avoid spoilage. Nuciferine (83.2% nuciferine; Zerun Pharmaceutical Co., Ltd., Chaozhuo, China) was administered by gavage in 0.5% carboxymethyl cellulose (CMC) buffer solution. Hamsters in the ND and HFD groups were administered 0.5% CMC buffer only. Food consumption and weight gain were measured daily and weekly, respectively.

After 8 weeks of nuciferine supplementation, the hamsters were anesthetized with pentobarbital after a 12-h fast and blood samples were drawn from the inferior vena cava. Serum was obtained by centrifuging the blood at 1500 rpm for 15 min at 4°C. Then, the remaining blood in the entire circulation system was flushed with physiological saline by perfusion through the left ventricle. Liver, epididymal adipose, and perirenal adipose tissues were excised, rinsed with physiological saline, weighed, flash frozen in liquid nitrogen, and stored at −80°C until assayed.

### Serum Parameter Measurements

Serum triglyceride (TG), total cholesterol (TC), high-density lipoprotein cholesterol (HDL-C), low-density lipoprotein cholesterol (LDL-C), free fatty acid (FFA), and glucose levels were determined using enzymatic kits purchased from Zhongsheng Beikong Biological Technique Company (Beijing, China) following the manufacturer’s instructions. The serum malondialdehyde (MDA) concentration and activities of superoxide dismutase (SOD), glutathione peroxidase (GPx), and catalase (CAT) were measured using assay kits purchased from Nanjing Jiancheng Bioengineering Research Institute (Nanjing, China). Enzyme linked immunosorbent assay kits for assessment of tumor necrosis factor-α (TNF-α) and interleukin-6 (IL-6) were obtained from 4A Biotech Co., Ltd. (Beijing, China). Serum β-hydroxybutyrate (β-HBA) levels were measured using an assay kit purchased from Co-Health Laboratories Co., Ltd. (Beijing, China). Serum uric acid and alanine aminotransferase (ALT) were assayed using kits purchased from Roche Diagnostics (Shanghai, China). Serum concentrations of insulin, adiponectin (APN), and leptin were determined using the Linco Multispecies Radioimmunoassay Kit (Linco Research, Inc., St. Louis, MO, USA). Blood insulin resistance was estimated using the homeostasis model assessment for insulin resistance (HOMA-IR) derived from the following equation: HOMA-IR = fasting serum glucose level (mg/dL)× fasting serum insulin level (ng/mL/22.5).

### Hepatic Lipids and Histology

For histopathological analysis, liver tissues were placed in formalin for no more than 24 h and then fixed in neutral-buffered formalin solution, dehydrated in graded alcohol, cleared in xylene, and embedded in paraffin. Then, these blocks were sectioned using a microtome, dehydrated in graded alcohol, embedded in paraffin, sectioned, and stained with hematoxylin and eosin for microscopic examination. The pathological changes were assessed by two independent liver pathologists using a fluorescence digital imaging microscope (BX-51; Olympus Corp., Tokyo, Japan). A semiquantitative scoring system was used to assess the severity of hepatic steatosis and inflammatory cell infiltration in 10 microscopic fields examined at 200× magnification as described previously [14], [15]. Briefly, liver tissues were scored for hepatic steatosis (0, none; 1, 1–25%; 2, 26–50%; 3, 51–75%; and 4, 76–100% hepatocytes affected) and necroinflammation (0, no inflammation; 1, mild lobular/portal inflammation; 2, moderate lobular/portal inflammation; 3, severe lobular/portal inflammation).

Hepatic lipids were extracted using the method described by Folch et al. [16] with slight modifications. Briefly, 100 mg of frozen liver tissue (stored at –80°C) was thawed and then homogenized for 5 min in 1 mL of ice-cold phosphate-buffered saline using a tissue homogenizer (T-10 basic Ultra-Turrax; IKA Werke GmbH & Co. KG, Staufen im Breisgau, Germany). A 2.0-mL aliquot of chloroform/methanol (2∶1, v/v) was added to the homogenate and vortexed for 60 s. After standing at 4°C in the dark for 12 h (hepatic lipids can be optimally extracted after this period), the homogenate was centrifuged at 2300×g for 15 min. The bottom organic phase was dried under nitrogen gas and the residue was suspended in 1 mL of 3% Triton X-100 and an aliquot was used for measurement of TG, TC, and FFA using the same commercial kits used for serum lipid analysis.

### Gene Expression Analysis

Total RNA was extracted from stored frozen livers from the different hamster groups using TRIzol reagent (Invitrogen, Carlsbad, CA, USA). Complementary DNA (cDNA) was synthesized using oligo Dt-Adaptor primers and AMV Reverse Transcriptase XL as recommended by the manufacturer (Dalian San Bao USA Inc., Arcadia, CA, USA).

The mRNA expression of fatty acid translocase (FAT/CD36) was determined by semiquantitative real-time polymerase chain reaction (RT-PCR) as described previously [13]. Messenger RNA levels of a number of other genes were assessed by real-time quantitative RT-PCR. Primers for amplification of each gene are listed in Table S1. All PCR reactions were performed in a total volume of 50 µL consisting of cDNA derived from 10 ng of total RNA, forward and reverse primers at a final concentration of 400 nM, and 25 µL of SYBR Green PCR Master Mix (Applied Biosystems, Foster City, CA, USA). PCR cycling parameters were as follows: denaturation for 10 min at 95°C followed by 40 cycles of denaturation at 15 s at 95°C followed by annealing and elongation at 60°C for 1 min. All mRNA levels were normalized to 18S rRNA values and the results expressed as fold changes of the threshold cycle (Ct) value relative to controls using the 2^−ΔΔCt^ method [17]. To ensure amplification specificity during RT-PCR, amplified products were subjected to agarose gel electrophoresis to visually confirm the presence of a single amplicon of the expected size.

### Western Blot Analysis

Liver samples were homogenized for 30 min in ice-cold radioimmune protection assay lysis buffer (50 mM Tris, pH 7.4, 150 mM NaCl, 1% Triton X-100, 1% sodium deoxycholate, 0.1% sodium dodecyl sulfate, 1 mg/mL leupeptin, 50 mM sodium fluoride, 1 mM sodium orthovanadate, and 1 mM phenylmethylsulfonyl fluoride). Liver homogenates were then centrifuged at 12,000 rpm for 30 min at 4°C and protein concentrations were determined using the Bradford method. Equal protein amounts were separated by sodium dodecyl sulfate polyacrylamide gel electrophoresis and electro-transferred onto polyvinylidene difluoride membranes, which were then blocked with 1% bovine serum albumin and probed with primary antibodies against sterol regulatory element-binding protein 1c (SREBP-1c; Santa Cruz Biotechnology, Santa Cruz, CA, USA), peroxisome proliferator-activated receptor-α (PPAR-α; Abcam, Cambridge, UK), fatty acid synthase (FAS; Novus Biologicals, Littleton, CO, USA), fatty acid translocase (CD36; Life Span Bioscience, Seattle, WA, USA) and β-actin (Santa Cruz Biotechnology). After washing, the membranes were incubated with secondary antibodies conjugated to alkaline phosphatase (AP) (goat polyclonal to rabbit IgG; Santa Cruz Biotechnology) for 60 min at 30°C. The signal was amplified by color development using the ProtoBlot II AP System with a stabilized substrate (Promega Corporation, Madison, WI, USA). Data are presented as the ratio of the target protein to β-actin. Immunolabeled bands were quantified by densitometry and representative blots are shown.

### Statistical Analysis

All data are presented as mean ± standard deviation (SD). Statistical analyses were performed using GraphPad Prism statistical software (version 5.01; GraphPad Software Inc., La Jolla, CA, USA) on untransformed data. Significant differences among groups were determined by one-way analysis of variance followed by post-hoc multiple comparison tests. The Kruskal–Wallis test with Dunn’s multiple comparison post-test was used for the analysis of the degree of liver steatosis and inflammation. A probability (*p*)-value <0.05 was considered statistically significant.

## Results

### Effects of Nuciferine on Body Weight, Liver and Visceral Fat Weight, and Food and Energy Intake

The 6-week-old male golden Syrian hamsters fed a HFD for 8 weeks gained significantly more weight than those fed a ND and nuciferine supplementation (15 mg/kg·BW/day) significantly reduced final BW. The daily food intake did not differ among the experimental groups and daily energy intake of groups HFD, HFNL (low dosage nuciferine group; HFD plus nuciferine at 10 mg/kg•BW/day), and HFNH (high dosage nuciferine group; HFD plus nuciferine at 15 mg/kg•BW/day) were significantly higher than that in group ND. The relative weights of liver, epididymal adipose tissue, and perirenal adipose tissue (g/100 g BW) were significantly lower in nuciferine-supplemented groups than those in the HFD group (Table 1).

**Table 1:** Effects of nuciferine on body weight, liver and visceral fat weight, and food and energy intake.

	ND	HFD	HFNL	HFNH
Final body weight, g	136.4±8.7a	155.4±10.7b	147.1±8.9bc	142.7±8.8ac
Liver weight, g/100 g BW	3.42±0.47a	4.41±0.50b	3.80±0.41a	3.52±0.42a
Epididymal WAT, g/100 g BW	1.66±0.25a	2.41±0.29c	2.01±0.36b	1.83±0.22ab
Perirenal WAT, g/100 g BW	0.85±0.12a	1.19±0.24b	1.01±0.20a	0.93±0.17a
Food intake, g/day	8.12±0.27	7.99±0.28	7.85±0.35	7.92±0.27
Energy intake, kcal/day	31.51±1.05a	34.83±1.23b	34.23±1.53b	34.54±1.19b

Values are means±SD, n = 10 hamsters in each group. ND: normal diet group, HFD: high-fat diet group, HFNL: low dosage nuciferine group (HFD+10 mg/kg·BW/day), HFNH: high dosage nuciferine group (HFD+15 mg/kg·BW/day). a, b, c: Means in the same row with different online letters differ significantly, p<0.05.

### Effects of Nuciferine on Serum and Hepatic Lipids

**Figure 1:**
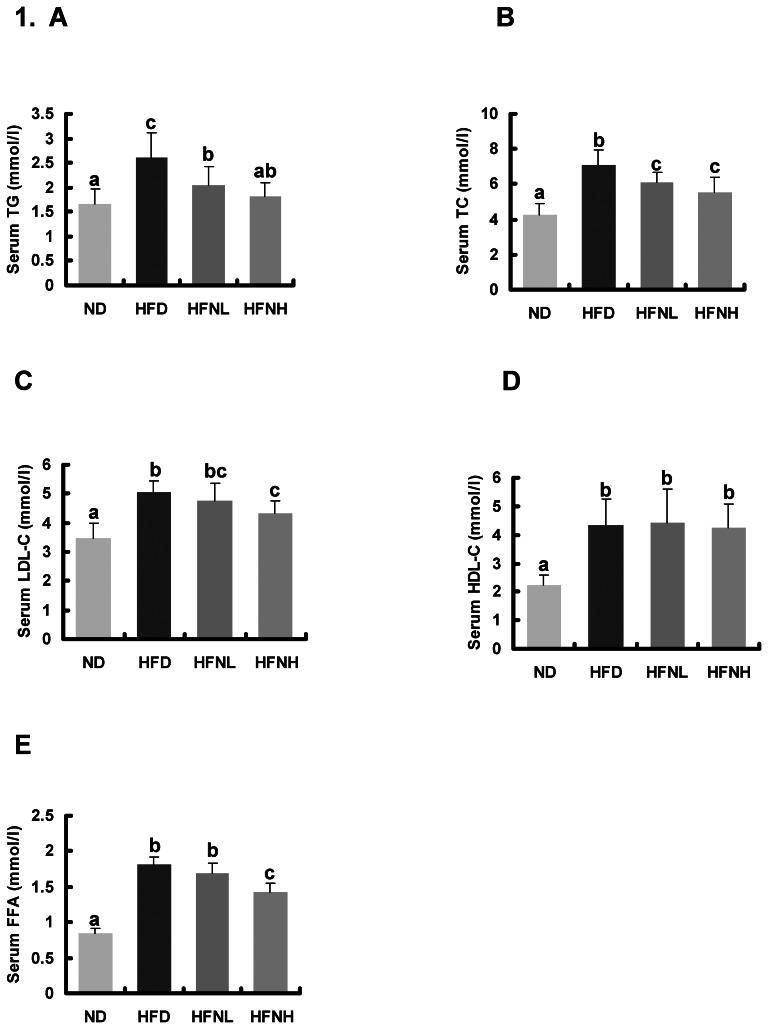
Effects of nuciferine on serum lipid profile and free fatty acid. (A) Serum triglyceride, (B) serum total cholesterol, (C) serum low-density lipoprotein cholesterol, (D) serum high-density lipoprotein cholesterol, and (E) serum free fatty acid. Values are means±SD, n = 10 hamsters in each group. ND: normal diet group, HFD: high-fat diet group, HFNL: low dosage nuciferine group (HFD+10 mg/kg·BW/day), HFNH: high dosage nuciferine group (HFD+15 mg/kg·BW/day). a, b, c: Means in the same row with different online letters differ significantly, p<0.05.

**Figure 2:**
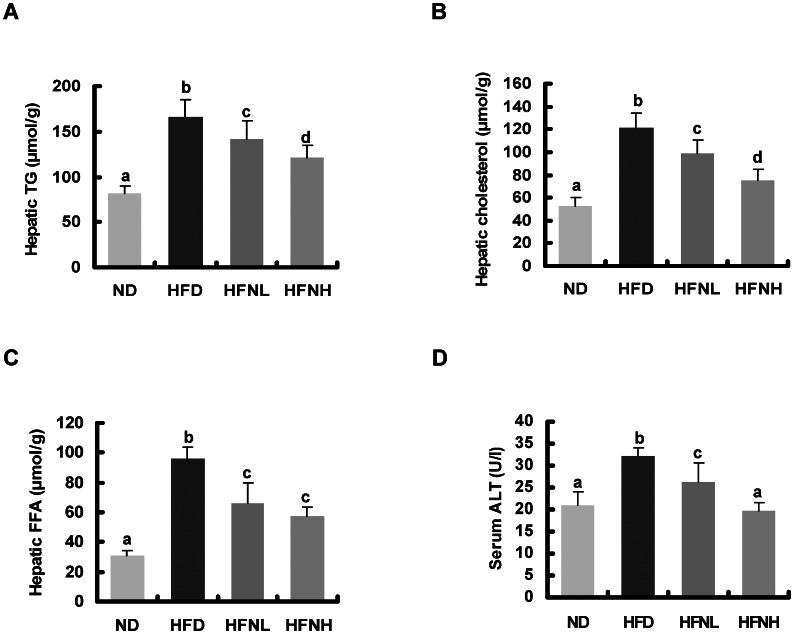
Effects of nuciferine on hepatic lipids and serum activities of ALT. (A) hepatic triglyceride, (B) hepatic cholesterol, (C) hepatic free fatty acid, (D) serum activity of ALT. Values are means±SD, n = 10 hamsters in each group. ND: normal diet group, HFD: high-fat diet group, HFNL: low dosage nuciferine group (HFD+10 mg/kg·BW/day), HFNH: high dosage nuciferine group (HFD+15 mg/kg·BW/day). a, b, c, d: Means in the same row with different online letters differ significantly, p<0.05.

Hamsters fed a HFD showed significantly higher serum levels of TC, TG, LDL-C, and FFA compared to those fed a ND, but nuciferine supplementation significantly reversed the HFD-induced elevation in serum lipid concentrations (Figure 1). HFD-induced elevation in serum ALT activity was significantly reversed by nuciferine supplementation. The hepatic accumulation of HFD-induced TG, TC, and FFA was also significantly alleviated by nuciferine supplementation (Figure 2). These results indicated that nuciferine supplementation significantly improved serum and hepatic lipid levels in the experimental hamsters.

### Hepatic Histology

**Figure 3:**
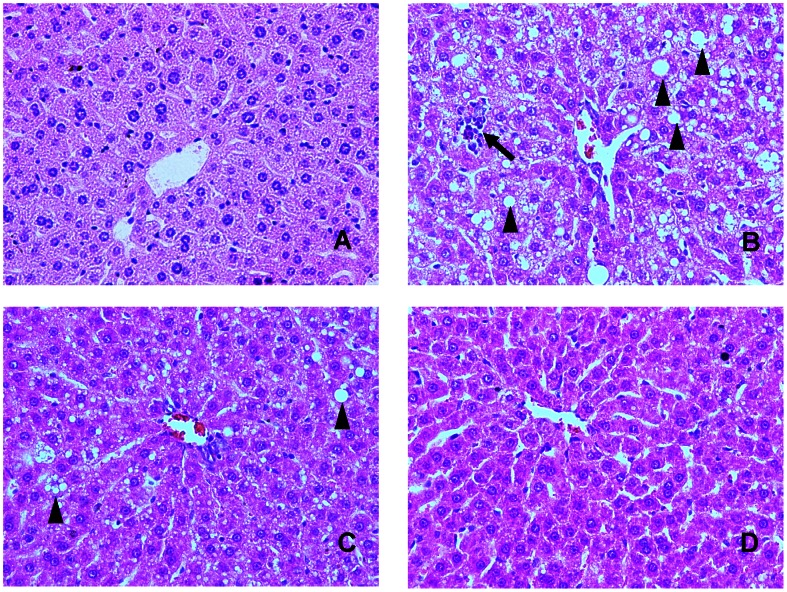
Effects of nuciferine on liver histology. (A) Liver sections from hamsters fed the normal diet showed normal liver histology. (B) In HFD fed hamsters, numerous macrovesicular fat droplets (arrow head) and mild necroinflammatory foci (arrows) were present. (C) and (D) Treatment with nuciferine (10 and 15 mg/kg·BW/day) for 8 weeks resulted in prevention of hepatic fatty deposition in hepatocytes and infiltration of the inflammatory cells in the hepatic parenchyma. The tissues were surgically excised and subjected to histological study by staining with hematoxylin and eosin. All the magnification is 200×.

Representative histological photomicrographs of liver specimens are shown in Figure 3. Hamsters fed a ND had normal liver histological findings; however, numerous macrovascular fat droplets and mild necroinflammatory foci were present in livers of those fed a HFD. Nuciferine treatment clearly improved hepatic steatosis and reduced hepatic necroinflammation. Especially, nuciferine at a dose of 15 mg/kg·BW/day effectively blocked necroinflammation development. Histological grading of liver sections confirmed that nuciferine supplementation significantly ameliorated both hepatic steatosis and necroinflammation (Table 2).

**Table 2:** Effects of nuciferine on scores for hepatic steatosis and necroinflammation.

	ND	HFD	HFNL	HFNH
**Steatosis**	0.0±0.0a	3.1±0.6b	1.9±0.7bc	1.3±0.5ac
**Necroinflammation**	0.0±0.0a	0.8±0.4b	0.5±0.5ab	0.2±0.4a

The severity of hepatic steatosis and necroinflammation were scored as following: In brief, liver tissues were scored for hepatic steatosis (0, none; 1, 1–25%; 2, 26–50%; 3, 51–75%; and 4, 76–100% hepatocytes affected) and necroinflammation (0, no inflammation; 1, mild lobular/portal inflammation; 2, moderate lobular/portal inflammation; 3, severe lobular/portal inflammation). Values of hepatic steatosis and necroinflammation are expressed as the means±SD, n = 10 hamsters in each group. The Kruskal–Wallis test with Dunn’s multiple comparison post-test was used for the analysis of the degree of liver steatosis and inflammation. ND: normal diet group, HFD: high-fat diet group, HFNL: low dosage nuciferine group (HFD+10 mg/kg·BW/day), HFNH: high dosage nuciferine group (HFD+15 mg/kg·BW/day). a, b, c: Means in the same row with different online letters differ significantly, p<0.05.

### Effects of Nuciferine on Serum Biochemistry

Serum levels of the proinflammatory adipokines TNF-α and IL-6 were significantly increased in hamsters fed a HFD. The HFD group also had significantly decreased serum activities of SOD, CAT, and GPx, and increased serum MDA concentrations. In addition, HOMA-IR and serum leptin and insulin concentrations were significantly elevated in the HFD group and serum APN and β-HBA levels were significantly decreased. Nuciferine supplementation significantly decreased HOMA-IR and serum levels of TNF-α, IL-6, MDA, leptin, and insulin, and increased serum APN levels and the activities of SOD, CAT and GPx. However, there were no differences in serum levels of glucose and uric acid among the groups (Table 3).

**Table 3:** Effects of nuciferine on serum biochemical parameters in high-fat-fed hamsters.

	ND	HFD	NFNL	NFNH
***Inflammation***				
TNF-α (pg/ml)	169.1±12.3a	236.1±28.3b	210.5±26.1b	166.2±11.4a
IL-6 (pg/ml)	466±77.6a	657.6±55.4b	613.2±81.4b	481.1±56.9a
***Oxidative stress***				
SOD (U/ml)	54.9±8.7a	40.9±3.6b	46.5±7.00b	60.6±9.7a
CAT (U/ml)	3.70±0.47a	1.52±0.20b	2.82±0.52c	4.38±0.77a
GPx	748.5±68.2a	649.6±81.1b	690.5±60.6ab	840.6±95.3c
MDA (nmol/l)	4.22±0.54a	6.59±0.79b	5.81±0.70c	5.49±0.71c
***Insulin resistance***				
Adiponectin (pg/ml)	120.3±21.4a	86.7±6.8b	91.2±7.3b	109.8±9.7a
Leptin (pg/ml)	313.4±25.9a	416.7±32.4b	387.6±40.2c	345.1±19.1d
Insulin (ng/ml)	0.76±0.08a	1.41±0.10b	1.36±0.10b	1.05±0.14c
Glucose (mg/dl)	86.35±13.98	86.94±14.10	89.32±9.41	85.00±13.05
HOMA-IR	2.92±0.56a	5.41±0.68b	5.39±0.63b	3.96±0.78c
***Others***				
β-HBA (µmol/l)	1141.7±149.3a	895.7±123.1b	1150.0±202.8a	1547.0±214.3c
Uric acid (µmol/l)	70.3±12.7	67.3±17.4	73.4±11.4	70.8±12.0

Values are means±SD, n = 10 hamsters in each group. ND: normal diet group, HFD: high-fat diet group, HFNL: low dosage nuciferine group (HFD+10 mg/kg·BW/day), HFNH: high dosage nuciferine group (HFD+15 mg/kg·BW/day). a, b, c, d: Means in the same row with different online letters differ significantly, p<0.05.

### Expression of Hepatic Genes Related to Lipogenesis

RT-PCR was used to assess the effects of nuciferine on expression levels of hepatic genes involved in lipid metabolism, including liver X receptor-α (LXR-α), sterol regulatory element-binding protein 1c (SREBP-1c), acetyl CoA carboxylase (ACC), fatty acid synthase (FAS), stearoyl-CoA desaturase 1 (SCD-1), and acyl-CoA:diacylglycerol acyltransferase 2 (DGAT-2). The results showed that expression levels of LXR-α, SREBP-1c, ACC, FAS, SCD-1, and DGAT-2 were significantly increased in the HFD group compared to the ND group and nuciferine supplementation significantly inhibited mRNA expression of these genes except DGAT-2 (Table 4).

**Table 4:** Effects of nuciferine on hepatic mRNA expression involved in lipid metabolism.

	ND	HFD	NFNL	NFNH
***Lipogenesis***				
SREBP-1c	1.00±0.08a	2.72±0.33b	2.25±0.19b	1.55±0.25c
LXR-α	1.00±0.07a	3.22±0.34b	2.39±0.37c	1.71±0.25d
ACC	1.00±0.11a	1.73±0.08b	1.43±0.15c	1.32±0.05c
FAS	1.00±0.13a	2.57±0.28b	2.04±0.24b	1.44±0.07c
SCD-1	1.00±0.08a	1.91±0.32b	1.35±0.18c	1.32±0.23c
DGAT-2	1.00±0.06a	3.45±0.69b	3.52±0.39b	3.65±0.42b
***FFA β-oxidation***				
PPAR-α	1.00±0.08a	0.66±0.04b	0.93±0.05a	1.24±0.13c
CPT-1	1.00±0.10a	0.73±0.05b	1.24±0.09c	1.40±0.09c
ACO	1.00±0.06	1.15±0.13	1.02±0.08	0.97±0.09
***FFA infiltration and VLDL secretion***				
PPAR-γ	1.00±0.10a	3.15±0.32b	2.63±0.19bc	2.30±0.33c
LPL	1.00±0.09a	6.98±0.90b	3.68±0.32c	2.70±0.45d
CD36	1.00±0.05a	3.62±0.27b	2.13±0.26c	1.55±0.13d
ApoB	1.00±0.14a	0.49±0.03b	0.86±0.06ac	1.26±0.16d
MTP	1.00±0.10a	0.39±0.05b	0.42±0.07b	0.86±0.12a
***Inflammation and Oxidation stress***				
TNF-α	1.00±0.08a	4.12±0.87b	3.05±0.52c	2.85±0.36c
CYP2E1	1.00±0.12a	2.69±0.16b	2.45±0.37b	1.53±0.06c

Specific mRNA values were normalized to the expression of 18S rRNA as described in Materials and Methods. Values are expressed as the means±SD (n = 4∼6) relative to the values obtained in hamsters fed the normal diet at 8 weeks, which were arbitrarily assigned a value of 1.00. ND: normal diet group, HFD: high-fat diet group, HFNL: low dosage nuciferine group (HFD+10 mg/kg·BW/day), HFNH: high dosage nuciferine group (HFD+15 mg/kg·BW/day). a, b, c, d: Means in the same row with different online letters differ significantly, p<0.05.

### Expression of Hepatic Genes Related to Fatty Acid β-oxidation

Next, we analyzed the mRNA levels of genes involved in fatty acid β-oxidation. Hamsters fed a HFD had significantly decreased expression levels of peroxisome proliferator-activated receptor-α (PPAR-α) and carnitine palmitoyltransferase 1 (CPT-1). Compared with hamsters fed a HFD, nuciferine supplementation significantly enhanced expression levels of PPAR-α and CPT-1. However, the expression levels of acyl-CoA oxidase (ACO) did not differ among groups (Table 4).

### Expression of Hepatic Genes Related to FFA Infiltration and VLDL Secretion

Next, we detected mRNA expression levels of genes related to FFA infiltration and VLDL secretion. The expression levels of peroxisome proliferator-activated receptor-γ (PPAR-γ), lipoprotein lipase (LPL), and FAT/CD36 were significantly increased in the HFD hamsters, but nuciferine supplementation significantly suppressed their expression. In contrast, expression levels of apolipoprotein B (ApoB) and microsomal triglyceride transfer protein (MTP) were significantly decreased in the HFD group, and nuciferine supplementation significantly enhanced their expression (Table 4).

### Expression of Hepatic Genes Related to Inflammation and Oxidative Stress

Feeding hamsters with a HFD for 8 weeks led to a significant up-regulation of the hepatic genes cytochrome P4502E1 (CYP2E1) and TNF-α, and the overexpression of these two genes was inhibited by nuciferine supplementation (Table 4).

### Nuciferine-induced Alterations in Levels of Hepatic Proteins Involved in Lipid Metabolism

**Figure 4:**
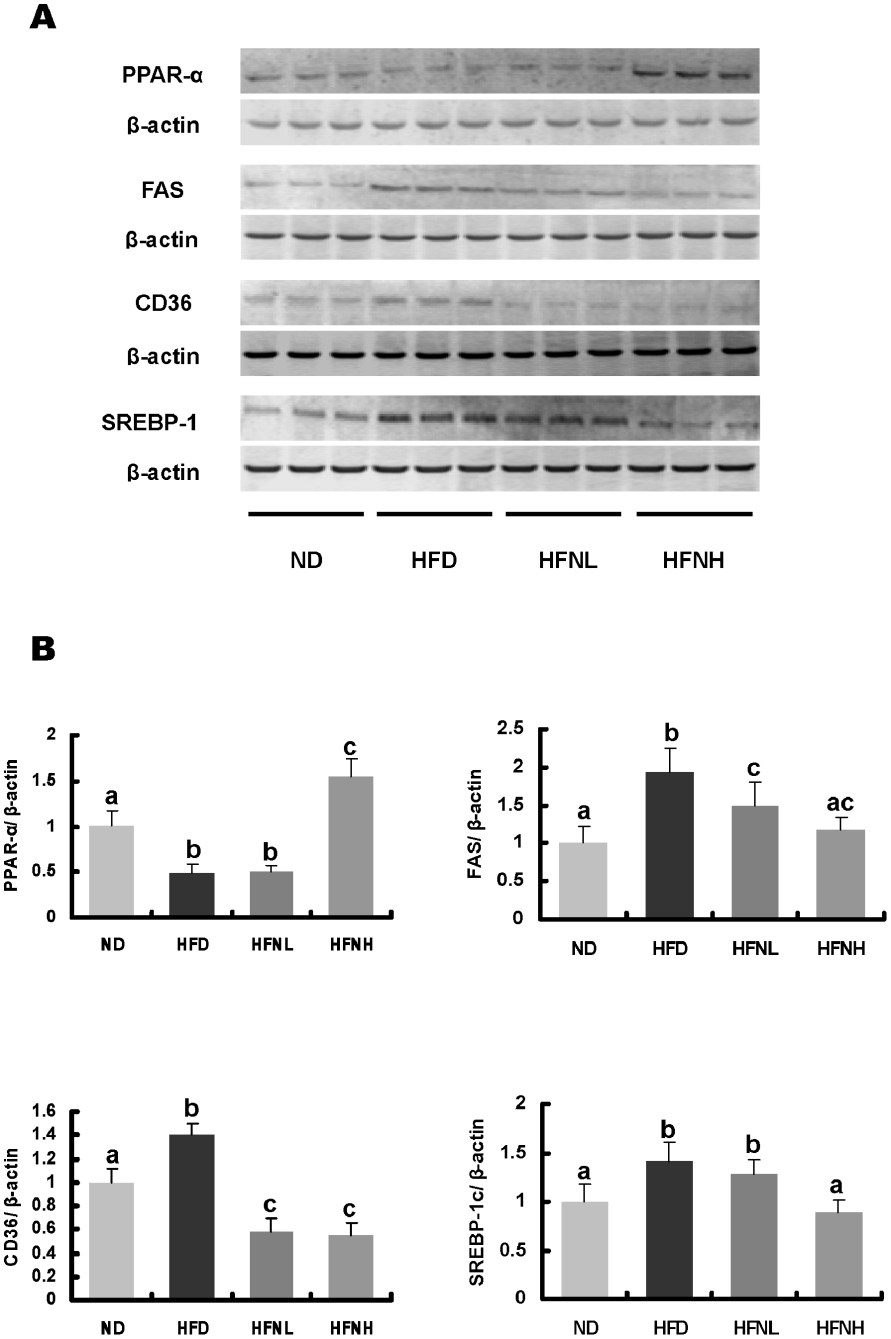
Effecs of nuciferine on hepatic protein levels of PPAR-α, CD36, FAS and SREBP-1c in high-fat fed hamsters. (A) Representative immunoblot showing the analysis of each protein. (B) Data graphed from image densitometric analysis of blots obtained from tissues of 10 separate animals (n = 10). ND: normal diet group, HFD: HFD group, HFNL: low dosage nuciferine group (HFD+10 mg/kg·BW/day), HFNH: high dosage nuciferine group (HFD+15 mg/kg·BW/day). a, b, c, d: Means in the same row with different online letters differ significantly, p<0.05.

In addition to mRNA levels, concentrations of a few select hepatic proteins were also measured (Fig. 4). We assessed hepatic levels of lipogenic proteins (SREBP-1c and FAS), as well as FAT/CD36, an enzyme involved in fatty acid uptake, and PPAR-α, which is involved in fatty acid oxidation. Hamsters fed a HFD had significantly increased hepatic protein levels of SREBP-1c, FAS, and CD36, and decreased hepatic PPAR-α levels. Nuciferine supplementation significantly reversed HFD-induced alterations in hepatic levels of these proteins (Figure 4).

## Discussion

NAFLD is perhaps the most common of all liver disorders and affects 10–40% of the population in several countries [18]. NAFLD is particularly linked to obesity, as the prevalence of steatosis is reportedly 57.5% and 74% in obese Japanese and Italian subjects, respectively [19]. The strong association of obesity with NAFLD suggests that obesity contributes to the pathogenesis of liver steatosis and progression to steatohepatitis; however, the pathophysiology leading to NAFLD is not well understood.

The hamster model of diet-induced liver steatosis with obesity and dyslipidemia was suggested as a good model for the study of human metabolic syndrome [20], [21], [22]. In the present study, hamsters fed a HFD had significantly increased body and liver weights, increased serum concentrations of TG, TC, LDL-C, FFA, and ALT, and liver concetrations of TG, TC, and FFA. On the contrary, nuciferine supplementation significantly decreased the body and liver weights, serum activity of ALT, as well as serum and liver contents of TG, TC, and FFA. In addition, nuciferine supplementation attenuated the development of HFD-induced hepatic steatosis and injury as assessed by microscopic analysis. These data demonstrated for the first time that nuciferine possesses biological activities in the regulation of lipid metabolism and steatosis development in the liver.

Reportedly, diet-induced obesity is associated with macrophage infiltration of white adipose tissue. Infiltrated macrophages, which are part of the stromal vascular fraction of adipose tissue, are subsequently responsible for the production of a wide variety of proinflammatory adipokines, including TNF-α and IL-6. Other than infiltrated macrophages, adipocytes also secrete a series of adipokines, such as APN and leptin, which are thought to play important roles in the regulation of metabolic homeostasis [23], [24]. Of the adipokines, TNF-α, IL-6, and leptin can induce IR [25]. In contrast, APN can improve IR and metabolic syndrome through its antiatherogenic, antidiabetic, and/or anti-inflammatory functions [26], [27]. In our study, HFD increased HOMA-IR and serum levels of TNF-α, IL-6, leptin, and insulin, and visceral adipose weight, while decreasing serum APN levels. Nuciferine supplementation contributed to the elevation of serum APN levels and the reduction of visceral fat weight, serum TNF-α, IL-6, leptin, and insulin concentrations as well as HOMA-IR. The above results indicated that nuciferine supplementation may ameliorate systemic IR by normalizing HFD-induced adipokine secretion.

IR with compensated hyperinsulinemia has been associated with steatosis and hyperlipidemia in humans and animal models [28]. Chronic hyperinsulinemia promotes hepatic lipogenesis through upregulation of lipogenic transcription factors, such as LXR-α, which is a nuclear transcription factor highly expressed in the liver that enhances transcription of several genes involved in lipogenesis, including SREBP-1c and its target genes ACC, FAS, SCD-1, and DGAT-2 in the presence of increased insulin [29], [30]. Normally, PPAR-γ is expressed at very low levels in the liver; however, in animal models of insulin resistance and fatty liver disease, its expression is markedly increased [31]. LPL and FAT/CD36 are PPAR-γ responsive genes, and their activation in the liver may increase FFA infiltration into the liver from blood [32]. In the current study, hamsters fed a HFD had increased protein levels of SREBP-1c and CD36, and upregulated mRNA expression levels of LXR-α, SREBP-1c, PPAR-γ, and their downstream target genes. These alterations were significantly reversed by nuciferine supplementation in hamsters fed a HFD, with the exception of DGAT-2. Therefore, we postulate that the improvement in liver steatosis in the nuciferine-supplemented group was partly caused by decreased expression of genes involved in lipogenesis.

Dysregulation of hepatic lipid metabolism may also improve through β-oxidation of lipids in the liver and the promotion of lipid discharge. Therefore, we examined PPAR-α and MTP gene and protein levels to evaluate lipid content and β-oxidation levels in the liver. CPT-1 and ACO are major enzymes that catalyze fatty acid β-oxidation and are responsive to PPAR-α activation. MTP is a heterodimeric lipid transfer protein present in the endoplasmic reticulum of hepatocytes and its activity is suppressed by insulin [33], [34]. MTP also plays an important role in VLDL assembly by mediating the transfer of hepatic lipids to nascent ApoB. Reductions in MTP activity and ApoB synthesis and secretion may impair hepatic lipid export and favor hepatic triglyceride accumulation [35]. Consistent with previous studies [34], we found that a HFD significantly decreased hepatic mRNA and/or protein expression levels of PPAR-α, CPT-1, MTP, and ApoB. In addition, the decreased concentration of serum β-HBA observed in the present study further indicated that hepatic β-oxidation and ketogenesis were indeed reduced in the HFD group. In contrast, nuciferine supplementation significantly increased serum β-HBA concentrations and hepatic mRNA and/or protein levels of PPAR-α, CPT-1, MTP, and ApoB. These results suggested that the beneficial effects of nuciferine in the treatment of liver steatosis may be partly due to enhanced FFA oxidation and lipid export in the liver.

Recent studies have demonstrated that lipid-induced oxidative stress plays important roles in NAFLD development [36]. CYP2E1 is a form of P-450, which is a member of the CYP2E gene superfamily and present in rodents and human. CYP2E1 induction has been also recognized as a major pathogenic feature of liver disease observed in both alcoholics and nonalcoholics. Fatty acid overload in hepatocytes acts as both a substrate and an inducer of CYP2E1 and its overexpression contributes to oxidative stress [37]. Consequently, oxidative stress causes the release of several cytokines, including TNF-α, a critical cytokine in hepatic steatosis development, by Kupffer cells [37]. MDA is a product of lipid peroxidation and can activate hepatic stellate cells, which play major roles in fibrogenesis in NAFLD. In our study, nuciferine supplementation significantly inhibited the overexpression of HFD-induced hepatic CYP2E1 and TNF-α mRNA levels. Nuciferine supplementation also increased serum antioxidant enzyme activities and decreased serum MDA concentrations. These results indicated that the beneficial effects of nuciferine supplementation on NAFLD may be partly attributed to its antioxidant activity.

Reportedly, nuciferine is structurally related to apomorphine and has a profile of action associated with dopamine receptor blockade. Intraperitoneal nuciferine administration at 25 mg/kg·BW in mice induced catalepsy and inhibited spontaneous motor activity, conditioned avoidance responses, amphetamine toxicity, and stereotypy [38]. However, no nervous effects were observed in hamsters in our study. It is well known that oral (p.o.) administration subjects a drug to potential first-pass elimination by the gut and liver, whereas the majority of an intraperitoneal (i.p.)-administered drug is absorbed into the hepatoportal circulation and only undergoes hepatic extraction. Therefore, different administration routes (i.p. vs. p.o.) and dosages (25 mg/kg vs. 15 mg/kg) might account for the absence of nervous effects in the current study.

Some limitations to the present study included the lack of suitable antibodies for hamsters and some important proteins were not investigated. The extrapolation of our data to other species and humans, however, remains complicated, thus further investigations are needed to match our results to those in studies using other species or humans.

In conclusion, the results of this study demonstrated, for the first time, that nuciferine exerted a protective effect on HFD-induced liver steatosis and injury through the modulation of lipid metabolism-related genes and/or proteins in hamsters. It is also possible that the anti-inflammation and anti-oxidation roles of nuciferine are integral to the protective functions of nuciferine in liver steatosis and its subsequent progression to steatohepatitis.

## Supporting Information

Table S1 **Primers used for the PCR reaction.**
(DOC)
