# Supplementary material for: Nuciferine Prevents Hepatic Steatosis and Injury Induced by a High-Fat Diet in Hamsters
Source: PLoS One. 2013 May 15;8(5):e63770. doi: 10.1371/journal.pone.0063770 (PMC3655021; doi:10.1371/journal.pone.0063770)
Supplement: Table S1 — Primers used for the PCR reaction. (DOC) [file pone.0063770.s001.doc]

**Table S1. Primers used for the PCR reaction.**

| **Gene** | **Sense and antisense** | **PCR product(bp)** | **Accesson number and Reference** | **Annealing temperature (℃)** |
| --- | --- | --- | --- | --- |
| SREBP-1c | 5’GCACTTTTTGACACGTTTCTTC3’  5’CTGTACAGGCTCTCCTGTGG3’ | 165 | U09103 | 60 |
| PPAR-α | 5’GTGGCTGCTATAATTTGCTGTG3’  5’AGCTTCGGGAAGAGAAAGGTAT3’ | 143 | AY170844 | 60 |
| PPAR-γ | 5’CACTCCCATTCCTTTGACATCA3’  5’AGCTGGGCCTTTTCAGAATAATAA3’ | 200 | Z30972 | 60 |
| LXR-α | 5’GCAACTCAATGATGCCGAGTT3’  5’CGTGGGAACATCAGTCGGTC3’ | 171 | U22662 | 60 |
| FAS | 5’GTGCTAGTGTCAACAAGCAATG3’  5’TTCAGGGTACCACTGTATTTGG3’ | 188 | AF356086 | 60 |
| SCD-1 | 5’CGTCAGCACCTTCTTGAGATAC3’  5’TCACTGGCAGAGTAGTCGTAGG3’ | 195 | J02585 | 60 |
| DGAT-2 | 5’TACAAGCAGGTGATCTTTGAGG3’  5’GGGCGAAACCAATATACTTCTG3’ | 79 | NM016384 | 60 |
| MTP | 5’ATTCAGCTGCAATCTGGACTAA3’  5’ACGAAAGAAGAGTCCACTGTGA3’ | 170 | U14995 | 60 |
| ACC | 5’ACACTGGCTGGCTGGACAG3’  5’CACACAACTCCCAACATGGTG3’ | 76 | AF356089 | 60 |
| ACO | 5’GCTGGCCGTGTCCATAGC3’  5’ TTATCCGTGGGTCCAAACTGA3’ | 63 | NM_030721 | 60 |
| 18S | 5’GTGGGCCTGCGGCTTAAT3’  5’GCCAGAGTCTCGTTCGTTATC3’ | 171 | NR_003278 | 60 |
| CPT-1 | 5’CTCAGTGGGAGCGACTCTTCA3’  5’GGCCTCTGTGGTACACGACAA5’ | 105 | NM_013495 | 60 |
| LPL | 5’CAGCTGGGCCTAACTTTGAG3’  5’CCTCTCTGCAATCACACGAA3’ | 215 | AF356087 | 60 |
| ApoB | 5’AGATGCCAACCTGGATTTCTTA3’  5’CCATATGGAGAAATCCTTCAGC3’ | 103 | M35187 | 60 |
| TNF-α | 5’CCCAAAGGGAAGAGAAGTTC3’  5’CCACTTGGTGGTTTGCTACA3’ | 126 | AF315292 | 60 |
| CYP2E1 | 5’GTGGTTGGGAATATAGGACA3’  5’CCAATAGCTCAGGCTTCTTA3’ | 111 | D17449 | 60 |
| CD36* | 5’CTTTGAGCCTTCCCTGTCTG3’  5’CAGCGTAGATGGATCTGAA3’ | 470 | U42430 | 60 |
| 18S* | 5’CGGTACAGTGAAACTGCGAAT3’  5’TCTGATAAATGCACGCATCC3’ | 156 | NR_003278 | 60 |

*Designed for semi-quantitative RT-PCR.

**References**

1. Gao S, He L, Ding Y, Liu G (2010) Mechanisms underlying different responses of plasma triglyceride to high-fat diets in hamsters and mice: roles of hepatic MTP and triglyceride secretion. Biochem Biophys Res Commun 398: 619-626.

2. Basciano H, Miller A, Baker C, Naples M, Adeli K (2009) LXRalpha activation perturbs hepatic insulin signaling and stimulates production of apolipoprotein B-containing lipoproteins. Am J Physiol Gastrointest Liver Physiol 297: G323-332.

3. Valeille K, Ferezou J, Amsler G, Quignard-Boulange A, Parquet M, et al. (2005) A cis-9,trans-11-conjugated linoleic acid-rich oil reduces the outcome of atherogenic process in hyperlipidemic hamster. Am J Physiol Heart Circ Physiol 289: H652-659.

4. Li G, Liu X, Zhu H, Huang L, Liu Y, et al. (2009) Insulin resistance in insulin-resistant and diabetic hamsters (Mesocricetus auratus) is associated with abnormal hepatic expression of genes involved in lipid and glucose metabolism. Comp Med 59: 449-458.

5. Lasa A, Simon E, Churruca I, Fernandez-Quintela A, Macarulla MT, et al. (2011) Effects of trans-10,cis-12 CLA on liver size and fatty acid oxidation under energy restriction conditions in hamsters. Nutrition 27: 116-121.

6. Zabala A, Churruca I, Fernandez-Quintela A, Rodriguez VM, Macarulla MT, et al. (2006) trans-10,cis-12 Conjugated linoleic acid inhibits lipoprotein lipase but increases the activity of lipogenic enzymes in adipose tissue from hamsters fed an atherogenic diet. Br J Nutr 95: 1112-1119.

7. Lowanitchapat A, Payungporn S, Sereemaspun A, Ekpo P, Phulsuksombati D, et al. (2010) Expression of TNF-alpha, TGF-beta, IP-10 and IL-10 mRNA in kidneys of hamsters infected with pathogenic Leptospira. Comp Immunol Microbiol Infect Dis 33: 423-434.

8. Choi MS, Lee MK, Jung UJ, Kim HJ, Do GM, et al. (2009) Metabolic response of soy pinitol on lipid-lowering, antioxidant and hepatoprotective action in hamsters fed-high fat and high cholesterol diet. Mol Nutr Food Res 53: 751-759.
